# AAV Vectors in Regenerative Medicine and Cellular Reprogramming: Potential, Pitfalls, and Specificity Constraints

**DOI:** 10.3390/ijms27156846

**Published:** 2026-07-30

**Authors:** Mariam Abdelnaby, Adelya Galiakberova, Erdem Dashinimaev

**Affiliations:** 1 Department of Biological and Medical Physics, Moscow Institute of Physics and Technology (State University), Institutskiy Per., 141701 Dolgoprudny, Russia; mariam.ak@phystech.edu; 2 Research Institute of Translational Medicine, Pirogov Russian National Research Medical University, Ostrovitianov Street, 117997 Moscow, Russia; adgaliakberova@gmail.com; 3 Research Institute of Molecular and Cellular Medicine, RUDN University, 117198 Moscow, Russia; 4 Medical Institute, Banzarov Buryat State University, 670000 Ulan-Ude, Russia

**Keywords:** adeno-associated virus (AAV), regenerative medicine, cellular reprogramming, gene therapy, cell-specific targeting, lineage tracing

## Abstract

The adeno-associated virus (AAV) has become the vector of choice for gene therapy and experimental gene delivery, owing to its non-pathogenic nature and ability to achieve persistent gene expression across diverse tissues. AAV has emerged as a key platform in cellular reprogramming and regenerative medicine, with applications spanning transcription factor delivery for in vivo lineage conversion and tissue repair across the CNS, heart, and musculoskeletal systems. However, significant limitations remain, particularly in the context of induced pluripotent stem cell (iPSC) engineering. We assess barriers to efficient iPSC transduction including receptor-dependent entry deficits and activation of p53-dependent DNA damage responses. Although AAV is widely described as non-integrating, evidence indicates that integration events occur in rapidly proliferating and actively reprogramming cells. Critically, we synthesize evidence that cell-type-specific promoters lose fidelity when paired with neurogenic transgene payloads, a cross-tissue problem not addressed in existing AAV reviews, and that published in vivo reprogramming efficiencies may be substantially confounded by promoter leakage in the absence of formal lineage tracing. These aspects, underrepresented in recent platform-level reviews, are specifically emphasized here as a resource for researchers designing rigorous AAV-based reprogramming and gene therapy strategies.

## 1. Introduction

**Figure 1:**
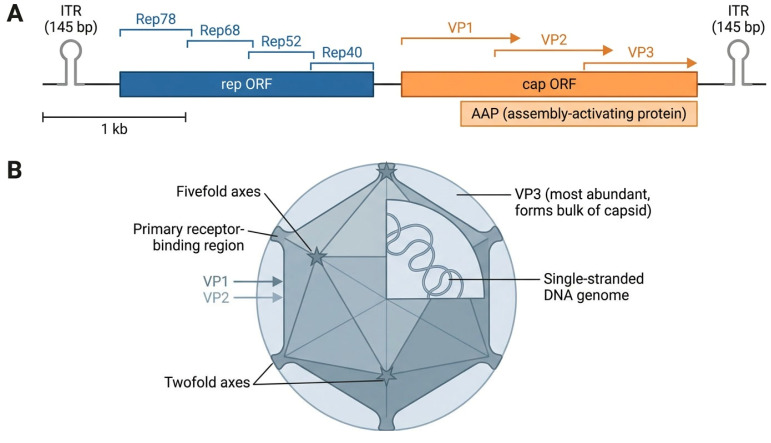
Schematic representation of the AAV genome organisation and capsid architecture. (**A**) Linear map of the 4.7 kb single-stranded DNA genome flanked by inverted terminal repeats (ITRs). The rep open reading frame encodes four non-structural proteins (Rep78, Rep68, Rep52, Rep40) and the cap ORF encodes the three capsid proteins (VP1, VP2, VP3) and the assembly-activating protein (AAP). (**B**) Cross-sectional view of the icosahedral AAV capsid (~25 nm) showing the distribution of VP1, VP2, and VP3, with threefold protrusions identified as the primary receptor-binding regions.

AAV has become a leading tool in clinical gene therapy owing to its low pathogenicity and capacity for long-term transgene expression across a wide range of tissues [1]. First identified in 1965 as a contaminant in adenovirus preparations, AAV has not been associated with any known human disease despite high seroprevalence in the general population [2]. The ~4.7 kb single-stranded DNA genome is flanked by inverted terminal repeats (ITRs) and encodes Rep proteins required for replication and integration, and capsid proteins (VP1–VP3) that determine tissue tropism and receptor binding [3,4] (Figure 1).

For gene therapy applications, the viral coding sequences are replaced with a therapeutic cassette to generate recombinant AAV (rAAV), retaining only the ITRs as cis-acting elements [5]. Unlike wild-type AAV, which integrates site-specifically at the AAVS1 locus via Rep-mediated recombination [6], rAAV vectors lack rep and persist predominantly as episomal concatemers, a property that substantially reduces insertional mutagenesis risk compared to integrating vectors and underlies the platform’s favorable safety profile. However, this non-integrating characterization is context-dependent: in rapidly proliferating and epigenetically remodeling cells such as iPSCs, integration events occur at frequencies that challenge the ‘integration-free’ label commonly applied to AAV, a limitation examined in detail in Section 6.6. AAV’s capacity for stable transgene expression in post-mitotic tissues, combined with its broad tropism and lack of known pathogenicity, has made it the dominant platform for both approved gene therapies and experimental reprogramming applications, though the conditions under which these advantages hold are more constrained than the field has generally acknowledged. Recent reviews have evaluated AAV within the broader gene delivery landscape or provided comprehensive overviews of its clinical applications [7,8,9,10]; however, three aspects central to cellular reprogramming remain under addressed in the existing literature and are specifically emphasized here. First, the iPSC-specific barriers to AAV transduction, including receptor absence, G-quadruplex-mediated cytotoxicity selectively in the pluripotent state, and epigenetic transgene silencing, are qualitatively distinct from barriers encountered in post-mitotic therapeutic contexts. Second, the insert-dependent failure of cell-type-specific promoters when paired with neurogenic transgene payloads has been documented across CNS, cardiac, and retinal tissues by multiple independent groups, yet has not previously been synthesized as a cross-tissue methodological problem. Third, this evidence supports the conclusion that a substantial proportion of published in vivo reprogramming efficiencies may be artefactual, and that mandatory lineage tracing should be adopted as a field-wide standard before such claims are accepted.

**Figure 2:**
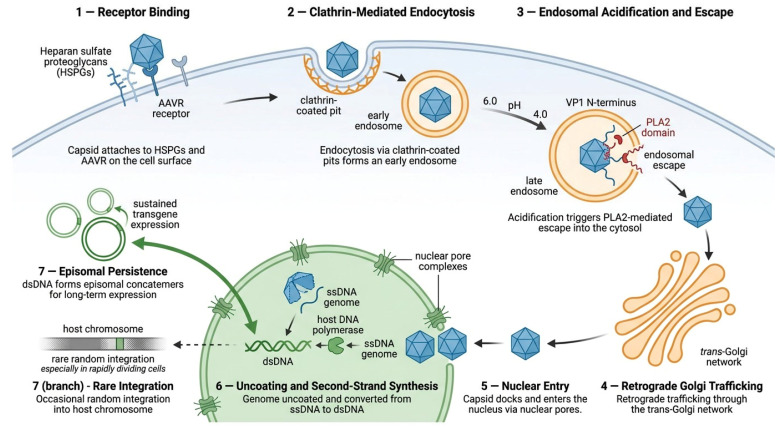
Schematic overview of the AAV intracellular life cycle, illustrated using AAV2 as the reference serotype. (1) AAV2 binds heparan sulfate proteoglycans (HSPGs) as a primary attachment receptor; other serotypes use distinct receptors including sialic acid (AAV1, AAV6), N-linked galactose (AAV9), or PDGFR (AAV5). The universal co-receptor *AAVR*/*KIAA0319L* is required for productive entry across multiple serotypes. (2) Internalization proceeds via clathrin-mediated endocytosis for AAV2; alternative endocytic routes have been reported for other serotypes. (3) Progressive endosomal acidification triggers conformational changes in the capsid, exposing the VP1 N-terminal PLA2 domain and enabling endosomal escape. (4) The virion undergoes retrograde trafficking via the trans-Golgi network before nuclear entry through the nuclear pore complex. (5) Inside the nucleus, uncoating releases the ssDNA genome, which is converted to dsDNA by host DNA polymerases. (6) In post-mitotic cells, the dsDNA circularizes and forms stable head-to-tail episomal concatemers that persist in the nucleus and support long-term transgene expression without integrating into the host genome—the basis of AAV’s favorable safety profile in non-dividing tissues. (7) In rapidly dividing or actively reprogramming cells, such as iPSCs, episomal genomes are passively diluted with each cell division due to their non-replicative nature; under chromatin-remodeling conditions associated with reprogramming, rare random integration events into the host chromosome can occur at frequencies sufficient to challenge the ‘integration-free’ characterization of AAV, as discussed in detail in Section 6.6. Arrow types in the figure indicate distinct biological processes: solid black arrows denote sequential steps of the canonical AAV2 intracellular trafficking pathway; curved or looping arrows in Step 6 indicate episomal circularization and concatemer formation in post-mitotic cells; the branching dashed arrow in Step 7 denotes the rare and stochastic event of chromosomal integration, which diverges from the predominant episomal persistence pathway and occurs at elevated frequency in actively dividing or reprogramming cells. Note: receptor usage, endocytic mechanism, and intracellular trafficking routes vary across serotypes; this figure depicts the AAV2 pathway as the best-characterized model and should not be interpreted as a universal description of AAV entry.

The intracellular journey from receptor binding through endosomal escape, Golgi trafficking, and nuclear entry to second-strand synthesis is summarized in Figure 2 and has been reviewed in detail elsewhere [7,8].

## 2. Molecular and Technological Foundations for Regeneration

One of the key technological advancements that allowed for the clinical translation of AAV was the invention of self-complementary AAV (scAAV) vectors. The packaging of the double-stranded, inverted-repeat genome of scAAV avoids the phase of second-strand synthesis of traditional single-stranded AAV, resulting in a 140-fold increase in transduction efficiency and earlier initiation of transgenic expression [11]. However, this gain comes at a direct structural cost: scAAV accommodates only ~2.5 kb of genetic cargo, roughly half the already limited capacity of single-stranded AAV. This renders scAAV unsuitable for the majority of therapeutically relevant transgenes currently in development, including full-length *dystrophin* (~11.4 kb) and *CEP290* (~7.5 kb). The efficiency advantage of scAAV is therefore not universally applicable but restricted to a narrow subset of small-cargo applications. The capacity constraints of both ssAAV and scAAV are discussed in detail in Section 7.

This clinical relevance is demonstrated by gene replacement therapy for spinal muscular atrophy type1 (SMA1), where systemic delivery of scAAV9 encoding human survival motor neuron (*SMN*) led to all treated infants surviving beyond 20 months, compared to only 8% in historical cohorts, and achieving previously unattainable motor milestones [12]. Similarly, a single peripheral infusion of scAAV expressing the coagulating factor IX in hemophilia B patients resulted in persistent therapeutic expression (2–11% of normal levels) changing a severe phenotype to a mild or asymptomatic one [13]. These investigations proved the clinical feasibility of AAV as a platform for sustained gene repair. Comparing these two trials reveals an important nuance: SMA1 therapy relied on systemic scAAV9 delivery in neonates—a population with immature immunity and an incompletely formed blood–brain barrier—while the hemophilia B results were obtained in adults with a targeted hepatic delivery strategy. The success of SMA1 therapy should therefore not be taken as straightforward evidence that high-dose systemic AAV is broadly safe in adult or pediatric populations; the immunological and physiological context of the patient is a critical variable that shapes both efficacy and risk.

## 3. Vector Engineering and Tissue Tropism

The wide biology of the capsid is closely related to the regeneration capacity of AAV. Different serotypes have different tissue tropism as the receptor binding and antigenicity are determined by the variations in the structure of surface exposed variable areas [14]. AAV9 has a strong tropism for the heart and CNS, while AAV8 is particularly successful in targeting the liver. In comparative studies, AAV9 has been demonstrated to provide long-term, high-level cardiac transduction with low off-target expression in mice and rats, making it especially well-suited for cardiovascular applications [15]. This serotype-dependent tropism is also observed at the cellular level: AAV1 and AAV6 have broad tropism for mammalian cell types, including human ESCs, whereas other serotypes have more restricted preferences such as AAV3 for human ESCs and AAV4 for murine adipose progenitors [16]. Table 1a summarizes the primary tissue tropism, receptor usage, and representative regenerative applications of these naturally occurring serotypes. It should be noted that the molecular mechanisms of cell entry also differ between serotypes: while clathrin-mediated endocytosis and HSPG-dependent attachment are well-characterised for AAV2, other serotypes utilise distinct primary receptors and may engage alternative endocytic pathways, for example, macropinocytosis has been implicated in AAV9 entry in certain cell types [7]. This mechanistic diversity is relevant to the interpretation of Figure 2, which depicts the AAV2 pathway as the best-characterised model and should not be taken as a universal description of AAV entry across serotypes. In a large-scale screening of 30 AAV variants in primary human cells and iPSCs, AAV7m8 proved highly efficient in human fibroblasts, T cells and iPSCs, AAV NP59 and NP40 performed well in human hepatocytes and AAV-LK03 and AAV3B were compatible with iPSCs at non-toxic doses [17].

Challenges such as pre-existing immunity and suboptimal targeting have been addressed through advanced engineering approaches, including directed evolution, and rational capsid design, to develop next-generation vectors with improved characteristics. Chimeric capsids, such as AAV-DJ, combine advantageous features from multiple serotypes, while targeted modifications, such as tyrosine-to-phenylalanine substitutions, improve intracellular trafficking and transduction efficiency at lower doses [14,18,19]. Recently identified naturally occurring variations such as AAVv66 further expand the toolset by increasing manufacturing yield, expanding CNS biodistribution and partially escaping neutralizing antibodies [20]. Table 1b presents the key engineered capsid variants discussed in this review, alongside their development strategy, primary target tissue, and principal performance characteristics. Together, the diversity of natural and modified serotype profiles govern the range of cell types that are susceptible to AAV-mediated transduction, a factor that is particularly relevant for the use of AAV vectors for reprogramming and directed differentiation. It is worth noting, however, that the most clinically advanced serotypes, AAV8 and AAV9, are also those with the broadest systemic biodistribution and the highest rates of liver sequestration. This reflects a historical tension in the field: vectors were selected for manufacturing yield and transduction potency long before specificity was systematically evaluated. The engineered variants identified in large-scale screens such as Westhaus et al. (2020) [17] offer improvements, but none has yet achieved the combination of high efficiency, narrow tissue specificity, and acceptable immunogenicity required for unrestricted clinical translation (Table 2).

**Table 1:** (**a**) Naturally Occurring AAV Serotypes in Regenerative Medicine. (**b**) Engineered AAV Capsid Variants.

(**a**)							
**Serotype**		**Primary Receptor**	**Target Tissue**	**Species Tested**	**Route**	**Key Finding**	**Reference**
**AAV1**		Sialic acid (α2-3, α2-6)	Inner ear; skeletal muscle	Mouse; NHP	Local (cochlear)	Restoration of near-normal hearing in *VGLUT3*-deficient mice; rapid IHC transduction	Akil et al., 2012 [21]
AAV2		HSPG; *FGFR1* (co-receptor)	Retina; liver; muscle	Mouse; human	Intravitreal; subretinal; IM	First-generation clinical vector; well-characterized but low efficiency in undifferentiated iPSCs	Summerford & Samulski, 1998 [22]
AAV5		PDGFR; sialic acid	CNS; lung; retina	Mouse; NHP	Intracranial; intranasal	Broad CNS distribution; 5.3-fold more vector DNA than AAV9 in cerebral organoids	Depla et al., 2020 [23]
AAV6		Sialic acid; HSPG	Cardiac muscle; lung; muscle	Pig; mouse	IV; IM	miR-199a delivery causing cardiomyocyte proliferation; 50% infarct size reduction	Gabisonia et al., 2019 [24]
AAV8		Laminin receptor	Liver; skeletal muscle	Mouse; dog; NHP; human	IV	High hepatocyte tropism; sustained Factor IX expression in haemophilia B	Nathwani et al., 2011 [13]; Koo et al., 2011 [25]
AAV9		N-linked galactose; *AAVR*	CNS; cardiac; systemic	Mouse; pig; NHP; human	IV; IT	Crosses BBB; SMA1 approval (Zolgensma); Dravet syndrome gene regulation	Mendell et al., 2017 [12]; Tanenhaus et al., 2022 [26]
(**b**)							
**Variant**	**Base Serotype**	**Engineering Strategy**	**Primary Target**	**Species Tested**	**Key Advantage**	**Key Limitation**	**Reference**
**PHP.eB**	AAV9	Directed evolution (CREATE)	CNS (broad, systemic)	Mouse (poor NHP efficacy)	>90% CNS neuron transduction after IV in mice	Species-specific receptor dependence; fails in NHP	Chan et al., 2017 [27]
**MyoAAV**	AAV9	Directed evolution	Skeletal muscle	Mouse (primate data limited)	Reduced liver sequestration vs. AAV9	6.3-fold elevated cardiac transduction vs. AAV9	Tabebordbar et al., 2021 [28]
**AAV2-7m8**	AAV2	Rational capsid modification	Retina (photoreceptors, RPE)	Mouse; iPSC organoids; NHP	64% photoreceptor transduction vs. 2.5% for AAV9	Receptor-dependent; *FGF2* inhibits via *FGFR1*	Garita-Hernandez et al., 2020 [29]
**rAAV.KK04**	AAV6	Directed evolution in hiPSC-CMs	Cardiomyocytes	Human cardiac slices; iPSC-derived	Outperforms AAV6 in 2D, organoids, and slices	High MOI amplifies DDR/*p53*/*STING*; not yet in vivo	Kok et al., 2023 [30]
**AAV-DJ**	Chimeric (AAV2/8/9)	Capsid shuffling	Multiple (primarily in vitro)	Human primary cells; iPSCs	High in vitro efficiency; heparin-binding domain	Broad tropism; limited specificity; research use	Drouin & Agbandje-McKenna, 2014 [14]
**AAVv66**	NHP isolate	Naturally occurring variant	CNS (broad)	Mouse; NHP	Improved yield; partial NAb evasion; broad CNS distribution	Limited clinical data	Hsu et al., 2020 [20]

Engineered variants are listed separately from naturally occurring serotypes to distinguish their development rationale and translational status. NHP, non-human primate; IV, intravenous; hiPSC-CM, human iPSC-derived cardiomyocyte; NAb, neutralising antibody; DDR, DNA damage response; RPE, retinal pigment epithelium; MOI, multiplicity of infection; CREATE, *Cre* recombination-based AAV targeted evolution.

## 4. Selected Regenerative Applications Across Organ Systems

The following section highlights representative AAV-based regenerative applications across CNS, musculoskeletal, sensory, cardiac, and wound-healing contexts. This is not an exhaustive survey of all organ systems; rather, the examples are selected to illustrate the range of AAV delivery strategies and the recurring translational challenges that arise across tissue types.

### 4.1. Gene Regulation and Nerve Regeneration

The limited internal repair capacity of CNS makes it a target for AAV-based regenerative methods. An AAV9-mediated gene therapy approach for Dravet syndrome demonstrated that targeted upregulation of endogenous SCN1A (encodes the alpha subunit of the voltage-gated type I sodium channel) using engineered transcription factors has the ability to restore neuronal function, reduce seizure frequency by 68%, and restore survival in treated mice [26]. Notably, therapeutic expression was maintained at long-term follow-up, consistent with stable episomal persistence. In addition to gene correction, AAV is being developed as a delivery platform for in vivo cellular reprogramming, where resident glial cells are converted into functional neurons. The use of transcription factors such as *Ascl1*, *Brn2*, and *Myt1l* has laid the foundation for studies that have shown that direct neuronal conversion can take place within the adult brain microenvironment, suggesting a potent strategy for replacing lost neurons in neurodegenerative diseases [33]. These approaches are particularly amenable to AAV-mediated delivery using CNS-tropic serotypes such as AAV9 and AAVrh10, which can cross the blood–brain barrier and achieve widespread neuronal transduction, positioning AAV as a promising platform for regenerative neurology [34].

### 4.2. Repair of Muscle and Structural Tissue

AAV delivery of engineered microdystrophin constructs has demonstrated robust therapeutic efficacy in skeletal muscle diseases such as Duchenne muscular dystrophy (DMD). In dogs, AAV2/8 delivery of codon-optimized microdystrophin restored dystrophin-associated protein complexes, attenuated fibrosis and necrosis and enhanced muscle integrity, without signs of immunological responses [25]. These findings underscore the importance of transgene optimization and the use of tissue-specific promoters in achieving durable and effective muscle regeneration.

### 4.3. Sensory and Cardiac Regeneration

AAV has also shown significant regenerative potential in sensory and cardiac tissues. Delivery of *VGLUT3* (Vesicular glutamate transporter 3) using AAV1 in a mouse model of inherited deafness resulted in restoration of near-normal hearing thresholds in two weeks, with recovery maintained for up to nine months [21]. AAV6-mediated delivery of miR-199a into the heart of infarcted pigs resulted in cardiomyocyte proliferation and approximately 50% reduction in infarct size, causing significant improvement in cardiac function. However, long-term expression of this microRNA leads to lethal arrhythmias, highlighting the essential need for fine temporal control of pro-regenerative signals [24].

### 4.4. Complex Biomaterials for Tissue Repair

Emerging strategies incorporate AAV into multicomponent regenerative systems, including biomimetic scaffolds designed to enhance wound healing. Extracellular vesicle-encapsulated AAV (EV-AAV) is an engineered delivery format in which AAV particles are packaged within lipid membrane vesicles, conferring partial shielding from neutralizing antibodies. When incorporated into hydrogel matrices, EV-AAV has demonstrated enhanced transduction efficiency and reduced immunogenicity compared to conventional AAV [35]. In diabetic wound models, VEGF-expressing EV-AAV combined with mesenchymal stem cell-derived exosomes promoted angiogenesis, modulated inflammation, and achieved complete wound closure within days [36]. This approach reflects a broader shift from single-gene interventions toward integrated regenerative platforms capable of addressing multiple interconnected components of tissue repair in parallel.

The integration of EV-AAV into biomaterial platforms represents a conceptual departure from conventional gene therapy, in which the vector is administered as a standalone agent. By embedding EV-AAV within a spatially defined scaffold, it becomes possible to restrict transduction to the anatomical site of injury, reducing systemic vector exposure and concentrating the gene delivery event at the tissue repair interface. This approach also allows for sequential or sustained release kinetics that are not achievable with bolus vector injection, potentially enabling the temporal control of transgene expression that single-dose AAV administration cannot provide. However, EV-AAV manufacturing presents additional complexity relative to conventional rAAV production, including the need to control exosome size distribution, encapsulation efficiency, and the ratio of genome-containing to empty EV-AAV particles, parameters that are not yet standardised. The combination of EV-AAV with mesenchymal stem cell-derived exosomes, as demonstrated by [36], further complicates the attribution of therapeutic effect to individual components of the platform, which is a challenge that will need to be addressed systematically before these approaches can be translated to clinical settings.

## 5. AAV-Mediated Reprogramming and Cell Engineering

In 2006, Shinya Yamanaka showed that forced expression of four transcription factors, *Oct4*, *Sox2*, *Klf4*, and *c-Myc*, reprograms somatic cells into induced pluripotent stem cells (iPSCs) [37]. These cells closely resemble embryonic stem cells in their ability to self-renew and give rise to multiple lineages, demonstrating that cell identity is not fixed but can be reset through progressive epigenetic changes in gene expression and chromatin organization [37,38,39,40,41].

Although iPSCs have great potential for personalized regenerative medicine and disease modeling [42,43,44], their generation is inefficient and stochastic and concerns regarding viral vector integration and oncogenic risk from c-Myc remain. To address these concerns, non-integrating delivery strategies have since improved the safety profile of iPSC-based approaches [45,46,47,48], and AAV vectors have emerged as a particularly promising platform for driving controlled reprogramming and directed differentiation across diverse cell types.

AAV is one of several non-integrating delivery strategies developed in response to the oncogenic risks associated with integrating retroviral vectors in iPSC generation. Episomal plasmid vectors, delivered by nucleofection, allow transient expression of reprogramming factors without viral components and have been used to generate clinical-grade iPSCs, but their reprogramming efficiency is lower than viral methods and they require multiple rounds of selection to eliminate residual plasmids [49]. mRNA-based delivery of reprogramming factors offers the most transient expression profile and avoids any nuclear DNA intermediate, but requires repeated daily transfections over 7–17 days and is sensitive to innate immune activation by exogenous RNA, which must be suppressed pharmacologically [50]. Protein-based delivery eliminates nucleic acid entirely but is limited by poor intracellular uptake and low reprogramming efficiency. AAV occupies a distinct niche among these alternatives: it achieves higher transduction efficiency than episomal plasmids in many primary cell types, requires fewer administrations than mRNA, and unlike Sendai virus (another common non-integrating method) does not require cytoplasmic RNA replication machinery. However, as detailed in Section 5, AAV’s claim to non-integration is less absolute than that of mRNA or protein delivery, and its immunogenicity and cytotoxicity in pluripotent cells introduce barriers that the other methods do not share.

### 5.1. Inducing Pluripotency from Somatic Cells

AAV vectors have shown the ability to reprogram somatic cells into induced pluripotent stem cells (iPSCs). In early studies, Weltner et al. (2012) demonstrated that recombinant AAV2 vectors encoding the Yamanaka factors (OCT4, SOX2, KLF4, and c-MYC) could reprogram mouse embryonic fibroblasts (MEFs) to iPSCs with canonical pluripotency markers (SSEA1, *Nanog*) and trilineage differentiation potential [51]. scAAV vectors were used to express OKSM factors for efficient in vitro reprogramming of MEFs and, importantly, to achieve in vivo reprogramming following systemic delivery of AAV8 vectors. PSCs were derived from multiple tissues, including blood and bone marrow, underscoring the systemic potential of AAV-mediated reprogramming. Importantly, this reprogramming was successful even without c-MYC, highlighting the flexibility of factor combinations required for successful reprogramming [52].

Yet the same study identified vector integration events in all derived iPSC clones, including insertions at cancer-associated loci, a finding that directly undermines the ‘integration-free’ rationale for choosing AAV over retroviral vectors and is examined in detail in Section 6.6.

### 5.2. Targeting CNS Cells and Neural Conversion with AAV

AAV vectors are increasingly used for cellular reprogramming due to their ability to efficiently deliver transcriptional regulators in vivo with relatively low toxicity and low immunogenicity [1,52,53]. Moreover, AAV systems have been extensively used for precise genomic manipulation of iPSCs and their derivatives, improving the safety and controllability of reprogramming strategies in multiple cell types and tissues [52,54].

A major application of AAV-mediated cell fate control is the generation of neural cell types. Flitsch and colleagues (2022) demonstrated that engineered AAV capsid variants (AAV7P2, AAV1P2, AAV8P2, AAV9P1) efficiently transduce induced neural stem cells (iNSCs), iPSC-derived radial glia-like neural precursor cells (RGL-NPCs), and astrocytes. *NGN2* overexpression in astrocytes caused rapid neuronal conversion with upregulation of neuronal markers (*TUBB3*, *MAP2*, *NeuN*) and downregulation of *GFAP*, and astrocytes acquired neuronal morphology within 2 weeks [55].

Alternative combinations of transcription factors have also been employed to show AAV-mediated reprogramming towards neuronal lineages. For instance, mouse fibroblasts have been directly converted into neurons through delivery of factors such as *ASCL1*, *BRN2* and *MYT1L* [56]. AAV-based strategies have demonstrated therapeutic benefits in the context of neural injury [57]. It has been shown that the astrocyte-tropic AAVShH19 variant carrying NeuroD1 could turn reactive astrocytes into neurons after spinal cord injury. Importantly, when combined with aligned fibrous scaffolds for localized vector delivery, this strategy promoted guided axonal regeneration across the lesion site and partial recovery of locomotor function in a rat hemisection model [57].

In parallel, capsid engineering has allowed broad and cell-type-specific targeting in the nervous system. AAV-PHP.eB and AAV-PHP.S, developed by Chan et al. (2017) [27], efficiently transduce the central and peripheral nervous systems, respectively, after systemic administration in mice. When combined with cell type-specific promoters, these vectors can support selective gene expression in defined neural populations, including neurons and glial cells [27]. This claim of “selective” expression warrants caution. As discussed in detail in Section 6, cell-type preference in the CNS is strongly modulated by administration, age, and transgene identity, not by capsids alone. PHP.eB achieves broad CNS transduction, but exclusivity to a single cell population has not been demonstrated. Furthermore, PHP.eB shows dramatically reduced efficiency in non-human primates compared to mice, limiting its translational relevance [31]. The apparent selectivity reported in initial murine studies has not held across species or experimental contexts.

Despite these advances, rigorous experimental controls are essential for the accurate interpretation of AAV-mediated neuronal reprogramming results. Puglisi et al. (2024) [58] demonstrated that retroviral delivery of a phospho-resistant Neurogenin2 induced a robust astrocyte-to-neuron conversion, as confirmed by lineage tracing. In contrast, AAV-FLEX delivery of the same factor resulted in reporter expression in pre-existing neurons, rather than genuine reprogramming [58]. Xie et al. (2023) [59] similarly showed that AAV-GFAP mediated expression of reprogramming factors (*NeuroD1*, *Math5*, *Ascl1* and *Neurog2*) can cause transgene-dependent leakage into endogenous neurons. This phenomenon was independent of serotype, route of delivery or viral dose, highlighting the need for rigorous lineage tracing to avoid overestimation of the efficiency of glia-to-neuron conversion [59].

### 5.3. Differentiation and Functional Engineering of Cells Derived from Pluripotent Stem Cells

Capsid screening in iPSC-derived neural models has identified engineered variants that substantially outperform natural serotypes. High-throughput screening of 30 AAV variants identified AAV7m8, AAV LK03, and AAV DJ as the most efficient for undifferentiated iPSC transduction [60], while parallel screening in iPSC-derived brain cells pinpointed peptide-engineered variants AAV7P2, AAV1P2, AAV8P2, and AAV9P1, which achieved transduction efficiencies of up to 98% in induced neural stem cells and demonstrated functional utility for shRNA-mediated knockdown and *NGN2*-driven neuronal differentiation [55].

Undifferentiated PSCs are generally less permissive to AAV transduction, owing to the activation of DNA damage response pathways triggered by the viral genome, as well as receptor-dependent barriers to entry and the absence of mature cell-surface receptors discussed elsewhere in this review [61]. However, their differentiated progeny is highly amenable to AAV-mediated genetic modification. For instance, human iPSC-derived cardiomyocytes (hiPSC-CMs) are efficiently transduced, in particular by cardiotropic serotypes such as AAV2 and AAV6 [61].

This has made it possible to enhance the function of PSC-derived cells. Li et al. (2023) demonstrated that extracellular vesicle-associated AAV6 and AAV9 vectors effectively deliver the *SERCA2a* (sarcoplasmic reticulum calcium ATPase 2a) gene to hiPSC-CMs and murine cardiomyocytes, resulting in improved calcium handling and underscoring the potential of AAV-engineered hiPSC-CMs as a platform for cardiac disease modeling and cell-based therapy [62]. These results demonstrate that AAV vectors are particularly useful for post-mitotic, lineage-committed cells, in which stable transgene expression can directly modulate cellular function. Directed evolution in hiPSC-derived cardiomyocytes produced rAAV.KK04, which consistently outperformed AAV6 in two-dimensional cultures, cardiac organoids, and human cardiac slices [30]. In iPSC-derived hepatocyte organoids, AAV6 and AAV8 displayed the highest transduction efficiency across multiple donors without hepatotoxicity, although donor-to-donor variability was identified as an important confounding factor in predicting human-specific responses [63].

### 5.4. Engineering Immune and Therapeutic Effector Cells

AAV vectors are also increasingly used to create engineered immune cell populations for therapeutic applications. Ye et al. (2024) [64] describe the MAJESTIC system (mRNA AAV–SB Joint Engineering of Stable Therapeutic Immune cells), which combines AAV-mediated delivery of a Sleeping Beauty transposon with mRNA-encoded transposase to enable stable genomic integration of chimeric antigen receptor (CAR) constructs. With AAV6, this platform enabled the generation of multiple types of engineered immune cells, including CAR-T cells, CAR-NK cells, CAR macrophages, CAR monocytes, and CAR-expressing iPSCs [64]. These results demonstrate the feasibility of AAV-mediated platforms for broad immune cell engineering workflows applicable to adoptive cell therapy.

### 5.5. Tissue-Specific Stem and Progenitor Cell Transduction and Engineering

In addition to neural and cardiac systems, AAV vectors have been successfully used to target tissue-resident stem cells. Konkimalla et al. (2023) [65] demonstrated that AAV5 transduced alveolar type II (AT2) stem cells in vivo following intranasal delivery, enabling lineage tracing and conditional gene inactivation. Furthermore, AAV6 efficiently transduced both murine and human AT2 cells in organoid cultures and was used to deliver CRISPR components for multiplex genome editing [65]. These findings reveal the potential of AAV vectors in lung stem cell biology, disease modeling, and regenerative medicine.

Collectively, the studies reviewed above establish iPSCs and their differentiated derivatives as versatile platforms for systematic capsid evaluation. Across neural, cardiac, retinal, and hepatic contexts, directed evolution and high-throughput screening have consistently identified engineered variants, including AAV2-7m8, rAAV.KK04, AAV7m8, AAV LK03, and the peptide-modified neural variants, that substantially outperform naturally occurring serotypes [29,30,55,60,66]. These findings underscore that serotype selection, vector dose, and capsid design must be optimized in a cell-type-specific manner rather than extrapolated from standard in vitro models. However, the same optimization strategies introduce their own constraints: the high multiplicities of infection required even by improved capsids further potentiate DNA damage responses through the p53/STING axis [67], and dose escalation as a compensatory strategy is limited by well-documented risks of hepatotoxicity and complement activation [68]. These trade-offs, together with the broader question of whether AAV reaches the right cells at all, are examined in the following section on specificity limitations. Taken together, these findings suggest that the iterative strategy of solving one AAV limitation through capsid engineering consistently unmasks another. Improved tropism demands higher MOI, which amplifies DDR. Dose escalation to compensate for inefficiency elevates toxicity risk. Specificity enhancements in one tissue increase off-target exposure in another. Rather than converging on a universal solution, the field appears to be navigating an interconnected set of constraints with no single engineering parameter that can be optimized without cost to another. This does not diminish the value of individual advances, but it does suggest that AAV-based reprogramming applications will require combinatorial strategies—including small-molecule DDR inhibitors, synthetic ITR variants, inducible promoter systems, and rigorous lineage tracing, rather than a single improved vector.

## 6. Specificity Limitations of AAV Vectors in Reprogramming Applications

A core challenge for AAV-based gene therapy and reprogramming across all tissue settings is the inability of current AAV vectors to achieve true cell-type specificity. This limitation manifests in two ways: at the level of capsid tropism, where systemic delivery leads to transduction of multiple off-target cell types and organs, and at the level of promoter specificity, where even supposedly cell-type-restricted promoters do not restrict transgene expression when paired with certain transgene payloads.

### 6.1. Broad Tropism and Liver Sequestration After Systemic Delivery

The predominant specificity challenge in AAV-based gene therapy is the broad hepatic tropism of most naturally occurring serotypes following intravenous administration. When delivered systemically, up to 90% of the viral payload is sequestered by the liver, rather than reaching the intended target organ, whether it is skeletal muscle, the heart, or the CNS [8,69], representing a major safety and efficacy limitation. For example, in muscle-directed gene therapy, natural capsid AAV9 vectors require doses approximately 100–250 times higher than engineered muscle-tropic variants to achieve equivalent therapeutic levels in skeletal muscle, largely as a consequence of hepatic sequestration after systemic administration [28].

Importantly, overcoming organ-level delivery barriers does not resolve the second major specificity challenge of AAV vectors: the inability to selectively transduce a single cell population within the target organ.

### 6.2. Lack of Cell-Type Specificity Within Target Organs

Even with organ-level targeting, AAV vectors transduce multiple distinct cell types simultaneously, a challenge with critical implications for reprogramming strategies that require cell-type-exclusive transgene delivery. The specificity problem goes even further within the liver: AAV vectors transduce not only the intended hepatocyte target but also non-parenchymal liver cells. In diseased and fibrotic liver, the distribution of AAV vector genomes is dramatically shifted toward endothelial cells (2.3-fold increase), Kupffer cells (5.3-fold), hepatic stellate cells (25.1-fold), and dendritic cells (7.2-fold) compared to healthy liver, fundamentally altering the cellular pattern of transgene delivery in pathological states, precisely the conditions in which liver-directed gene therapy is most needed [70]. In a healthy liver, even AAV8 carrying a hepatocyte-specific *TBG* promoter, a gold standard for hepatocyte targeting, results in off-target effects, including induction of DNA damage markers in non-hepatocyte cell populations and transient suppression of liver homeostatic proliferation, effects that are exacerbated when the vector expresses *Cre* recombinase [71].

In the CNS, no naturally occurring AAV serotype exists that yields exclusive glial or exclusive neuronal transduction. Comparative analysis of common variants (AAV1, AAV2, AAV6, AAV7, AAV8, AAV9, PHP.eB, PHP.S) in living human brain tissue showed that all tested capsids transduced multiple CNS cell types indiscriminately; most variants achieved 30–50% transduction across all cell types, and none achieved glial or neuronal exclusivity [72]. In adult mice, intravenous AAV9 preferentially targets astrocytes throughout the CNS, whereas the same vector delivered by direct intracranial injection exhibits a strong preference for neurons, indicating that the observed cell-type preference is a function of route of administration and age, not an intrinsic capsid characteristic [34]. Even serotypes with reported relative astrocyte preference, such as AAV5, transduced a majority of neurons (58% of transduced cells) when injected into brain parenchyma under pan-cellular promoters [73].

Intravenously administered AAV vectors targeting the myocardium simultaneously transduce cardiac fibroblasts and endothelial cells within the heart, as well as hepatocytes in the liver. The cardiac troponin T (*cTnT*) promoter confines transgene expression to cardiomyocytes, with a 640-fold enrichment over the next highest tissue, but vector genomes are physically present in non-cardiac cells and near 100% cardiomyocyte transduction efficiency requires doses that substantially expose the liver and other organs to vector particles [74]. This distinction is especially relevant in the context of cardiac reprogramming, where the goal is to convert fibroblasts into cardiomyocytes. AAV vectors expressing cardiac reprogramming factors under cardiomyocyte-specific promoters must not express these fate-determining transgenes in non-target cardiac cell populations, but the physical transduction of multiple cardiac cell types by the vector is unavoidable with current tools [75].

Cell-type specificity of AAV vectors in the retina is highly dependent on injection route and serotype combination and off-target transduction within the retina is ubiquitous. Intravitreal injection mainly transduces retinal ganglion cells, but also Müller glia and amacrine cells, with limited access to the photoreceptors and the retinal pigment epithelium because of the inner limiting membrane barrier [76]. In contrast, subretinal injection efficiently targets photoreceptors and RPE, but also non-specifically transduces multiple inner nuclear layer cell types, depending on serotype [77,78]. All studied capsid variants, including AAV7m8, AAVBP2 and AAV7m8(Y444F), revealed broad cellular transduction patterns in healthy and degenerated retinas in optogenetic therapy strategies specifically directed towards ON-bipolar cells and that the GRM6 promoter, which is commonly assumed to be ON-bipolar cell-specific, was in practice not specific [78,79].

In skeletal muscle, even next-generation engineered myotropic vectors such as MyoAAV, developed through directed evolution to overcome liver sequestration, resulted in increased muscle transduction and elevated cardiac transduction (6.3-fold higher than AAV9 in the heart), where increased specificity for one muscle compartment was achieved at the expense of increased off-target expression in another compartment [28]. The comparison of MyoAAV and AAVMYO variants demonstrated that the balance between liver detargeting and muscle enhancement depends on the age at injection, the dose and the genetic background of the mouse, none of the variants showing a uniform specificity for the muscular system without residual off-target distribution [32].

### 6.3. Reprogramming Contexts: Promoter-Specificity Failure

Even when the capsid tropism is partially controlled, specificity of transgene expression is further undermined at the level of the promoter, particularly in reprogramming applications. The short human *GFAP* minipromoter (GfaABC1D), designed to restrict transgene expression to *GFAP*-expressing astrocytes, fails when neurogenic transcription factors, such as *NeuroD1*, *Neurogenin2*, *Math5*, or *Ascl1*, are the transgene payload. This failure is not resolved by reducing viral dose, switching serotypes, or altering the route of injection [59,80].

**Figure 3:**
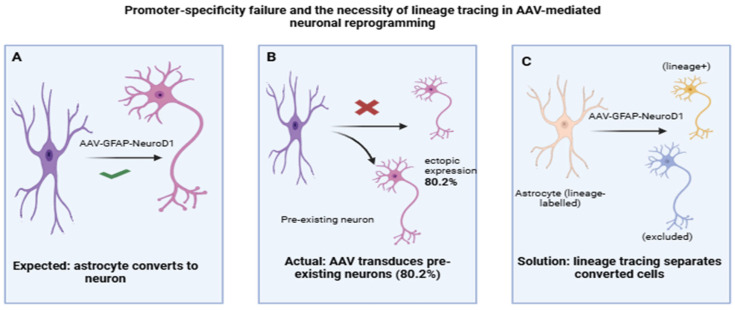
Promoter-specificity failure and the necessity of lineage tracing in AAV-mediated reprogramming. (**A**) Expected outcome: the *GFAP* minipromoter drives neurogenic transgene expression selectively in astrocytes, producing genuine astrocyte-to-neuron conversion. (**B**) Actual outcome: cis-regulatory interaction between the *GFAP* promoter and the neurogenic transgene payload causes ectopic expression in pre-existing endogenous neurons, generating false-positive reprogramming signals up to 80.2% of HuC/D-positive cells [59]. (**C**) Solution: Cre-lox lineage tracing permanently labels target cells prior to AAV delivery, enabling unambiguous identification of genuinely converted cells and exclusion of pre-existing neurons from the analysis. Solid arrows indicate the direction of transgene expression or cell fate conversion between cell populations. ✓ (checkmark) indicates confirmed or expected cell-type-specific expression in the intended target cell; ✗ (cross) indicates specificity failure.

The mechanism is a cis-regulatory interaction between the *GFAP* promoter and the transgene sequence, leading to ectopic expression in pre-existing neurons in up to 80.2% of HuC/D-positive cells, causing widespread false-positive reprogramming signals in multiple published studies [59] (Figure 3). Critically, this failure is insert-dependent: a control *GFAP*-mCherry construct was faithfully expressed in Müller glia, whereas five of seven transcription factor constructs under the same promoter were expressed predominantly in neurons rather than glia [80]. This insert-dependent promoter failure has been observed in various brain regions and verified by independent groups employing rigorous lineage tracing [58,59,80,81].

The first indication that the gfaABC1D minipromoter alone was not sufficient for astrocyte specificity was provided by Taschenberger et al., who demonstrated that a substantial proportion of transduced cells in the rat striatum were neurons even in the absence of a neurogenic transgene, and that the incorporation of miR-124 target sequences into the 3′ UTR partially reduced this leakage by exploiting the neuron-enriched expression of miRNA-124, but at the expense of lower overall transgene expression [82].

The specificity limitations described above extend beyond the context of astrocyte-to-neuron reprogramming. This principle—whereby cell-type-specific promoters lose fidelity depending on the transgene insert, host chromatin context, and AAV capsid—applies broadly across reprogramming applications in diverse tissue settings. For instance, in the heart, AAV-DJ vectors carrying a *periostin* promoter, intended to restrict expression to cardiac fibroblasts for in vivo reprogramming, showed strong and specific expression in resident fibroblasts after myocardial infarction. However, lineage tracing was required to confirm that transgene expression did not extend to cardiomyocytes, highlighting the need to empirically validate promoter-transgene cis-regulatory interactions for each new reprogramming factor and capsid combination [75].

In summary, the evidence presented here demonstrates that achieving true cell-type specificity with AAV vectors remains an unresolved challenge across multiple tissue contexts, cell types and therapeutic applications. Neither capsid engineering nor promoter choice alone is sufficient at present to ensure the expression of a transgene exclusively within a targeted cell type, and even the combined approach, despite its increased specificity, must be empirically validated through lineage tracing for each individual application.

### 6.4. Challenges in Transducing iPSCs Using AAV

Efficient AAV transduction of iPSCs is hindered by several barriers, including low surface expression of entry receptors, entry-related cytotoxicity, innate immune activation, passive dilution of episomal genomes with a concomitant risk of integration, and epigenetic silencing of delivered transgenes. AAV transduces undifferentiated iPSCs and hESCs approximately 100-fold less efficiently than their differentiated derivatives, a deficit that is independent of vector genome configuration and serotype [61]. Of the naturally occurring serotypes, AAV2 and AAV6 are consistently superior to AAV1 and AAV9 in iPSC-derived differentiated cells, while adenoviral vectors are superior in the undifferentiated state [61]. Reduced permissivity reflects the low surface expression of canonical AAV receptors on pluripotent cells, including sialic acids (AAV1/6), terminal N-linked galactose (AAV9), and the universal receptor *AAVR*/*KIAA0319L*, all of which are progressively upregulated during differentiation [9,61].

The receptor-dependent barrier has been well characterized in iPSC-derived retinal organoids. Garita-Hernandez et al. showed that the efficiency of transduction correlated directly with the presence of the corresponding cell-surface receptors at the time of infection for four serotypes (AAV2, AAV2-7m8, AAV8, AAV9) [29].

Another barrier to entry is that culture medium components such as *FGF2*, routinely used in retinal and RPE differentiation protocols, can competitively inhibit the AAV2 co-receptor *FGFR1*, reducing transduction efficiency in a time-dependent manner [29].

### 6.5. AAV-Triggered Cytotoxicity and DNA Damage Response (DDR)

AAV-mediated transduction can induce DNA damage response (DDR) and innate immune activation in a cell-state-dependent manner, with the strongest effects observed in pluripotent and progenitor populations.

*AAV vectors are not only inefficient in pluripotent cells but are also directly cytotoxic.* Rapti et al. reported substantial apoptotic cell death in several lines of hESCs and iPSCs within one to two days of AAV infection, mediated by upregulation of DDR and p53-pathway targets including *BTG2* and *p21*, while differentiated cardiomyocytes derived from the same lines remained unaffected [61]. The underlying cause is a G-quadruplex-forming sequence within the AAV ITR that mimics telomeric DNA and induces p53-dependent mitochondrial apoptosis selectively in the pluripotent state; empty capsids were not toxic, demonstrating that the encapsulated genome is the direct trigger [83]. Engineered synthetic ITR variants reduced this toxicity but at the cost of substantially diminished transgene expression, revealing a fundamental safety–efficacy trade-off [84].

The severity of AAV-induced cytotoxicity is inversely related to the degree of cellular maturation. In iPSC-derived retinal organoids, day-80 organoids containing proliferating progenitors exhibited morphological degeneration in 65–90% of cases following transduction with scAAV2, scShH10 and scAAV2.7m8, with increased caspase-3 cleavage and reduced *KI67* expression, while post-mitotic day-300 organoids were largely resistant [85]. Paradoxically, the DNA damage response is further potentiated in mature cardiac organotypic structures compared to two-dimensional iPSC-cardiomyocyte cultures. This means that the same maturation steps that improve physiological relevance simultaneously introduce new transduction barriers [30].

Even after differentiation to CNS lineages, the AAV genome alone induces an early p53-dependent DDR, followed by pro-inflammatory and type I interferon signaling, in iPSC-derived neurons, astrocytes and oligodendrocytes [67]. These signatures persist for up to five days in culture and 28 days in vivo. Small-molecule inhibitors of *p53* or *STING* prevented transduction-induced apoptosis in iPSC-derived neurons, representing promising mitigation strategies [67].

AAV transduction in hematopoietic stem and progenitor cells upregulates *p21* and pro-apoptotic genes such as *DHRS2*, *GZMB* and *GDF15*, as well as aberrant induction of *CD86* that may drive stem cells towards lineage commitment and decrease regenerative potential [86]. Additionally, non-productive infection events, in which vector genomes remain in the cytoplasm without nuclear entry, provide additional substrates for innate immune sensing via AIM2 or cGAS following proteasomal capsid degradation [86].

### 6.6. Risk of Episomal Dilution and Integration

rAAV genomes persist as non-replicative episomes and are therefore passively diluted with each cell division, a fundamental incompatibility with the rapid proliferation of iPSCs [9]. Weltner et al. showed that multiple rounds of serial transduction were required during reprogramming to maintain sufficient transgene expression, and that the majority of resulting iPSC clones harboured vector integration events under the chromatin-remodeling conditions of reprogramming [51]. Senís et al. also validated integration in all iPSC clones derived from in vivo scAAV8-based reprogramming, with 47% of the 180 identified integration sites being intragenic, including insertions within cancer-related gene loci [52]. These findings substantially challenge the ‘integration-free’ characterization of AAV that has been used to justify its selection over retroviral vectors in actively dividing cell contexts. This has practical consequences beyond experimental interpretation. Regulatory and ethical frameworks for AAV-based cell therapies have been built partly on the assumption of non-integration. If this assumption fails specifically in the cell populations most relevant to reprogramming, actively dividing and epigenetically remodelling cells, then the safety rationale for preferring AAV over lentiviral vectors in iPSC contexts becomes substantially weaker than the literature implies. A more honest framing would describe AAV as “low-frequency random integrating” in proliferating cells, rather than “non-integrating,” and future clinical applications in iPSC-derived therapies should incorporate integration site analysis as a standard safety endpoint.

### 6.7. Epigenetic Silencing of Introduced Transgenes

Even when productive transduction occurs, transgene expression in iPSCs is frequently silenced through CpG methylation of promoter sequences, including CMV, EF1α, CAG, and synthetic variants [87]. In the pluripotent state, silencing is largely irreversible and persists upon differentiation, including along neural lineages [87]. For example, CBX3-UCOE, a chromatin-opening regulatory element, has been shown to prevent methylation-dependent silencing in iPSCs and their differentiated derivatives, demonstrating that stable transgene expression requires careful engineering of regulatory elements beyond promoter selection alone [88].

## 7. General Limitations of AAV Vectors

Also, AAV vectors, despite their widespread use in the clinic, have basic biological and immunological limitations that limit their therapeutic usefulness (Table 3).

### 7.1. Capacity for Packaging

A principal limitation of AAV vectors is their restricted packaging capacity of approximately 4.7 kb of DNA, precluding the delivery of larger therapeutic transgenes [89]. This constraint is particularly relevant for genes such as full-length *dystrophin* (~11.4 kb) in Duchenne muscular dystrophy and *CEP290* (~7.5 kb) in Leber congenital amaurosis type 10, both of which substantially exceed this packaging threshold [90]. Self-complementary AAV (scAAV) vectors, designed to accelerate transgene expression by bypassing second-strand synthesis, accommodate only approximately 2.5 kb of genetic cargo, which is even less than single-stranded AAV vectors, and carry an elevated risk of immune responses owing to the rapid accumulation of transgene products [11,91]. Mitigation strategies include transgene truncation—such as mini- or microdystrophin constructs—and dual-vector approaches, in which two vector halves are co-delivered and reconstituted at the DNA, RNA, or protein level in vivo. However, these approaches increase the complexity of vector design and manufacturing [92,93].

### 7.2. Immunogenicity and Pre-Existing Immunity

A significant barrier to AAV-based therapy is the high prevalence of pre-existing immunity to AAV capsids in the human population. Human seroprevalence studies indicate that neutralising antibodies (NAbs) targeting various AAV serotypes are prevalent, with reported rates varying from under 10% to over 90%, contingent upon serotype and geographical region [94]. Pre-existing NAbs can inhibit effective vector transduction even at low titres [95]. Furthermore, cross-reactivity among serotypes complicates serotype-switching strategies intended to circumvent pre-existing immunity [96]. In addition to humoral immunity, AAV vectors elicit both innate and adaptive immune responses upon administration. Pattern recognition receptors, such as TLR9, TLR2, and MDA5/RIG-I, initiate innate immune responses upon detecting vector-associated molecular patterns [97,98]. Regarding the adaptive aspect, capsid-specific CD8^+^ T cell responses have been documented in gene therapy trials, accompanied by transaminitis and progressive loss of transgene expression [95,96,99].

These capsid-specific CD8^+^ T cell responses are dose-dependent, with the magnitude of the T cell response correlating directly with the administered vector dose [13,96].

Although these immunological challenges are most pronounced in systemic delivery, capsid immunogenicity remains a relevant consideration in serotype selection and formulating AAV-based reprogramming strategies, especially for translational applications [100].

Immunosuppressive co-administration can partially attenuate these responses but substantially increases the complexity and clinical risk of treatment protocols, particularly in the context of regenerative applications where repeated dosing over a patient’s lifetime may be required [100].

### 7.3. Dose-Dependent Toxicity

Dose-dependent toxicity represents one of the most clinically urgent limitations of AAV gene therapy, with serious adverse events documented across multiple high-dose trials [95].

High-dose systemic administration has been directly associated with serious adverse events: in the ASPIRO trial for X-linked myotubular myopathy, two patients receiving the highest vector doses developed fatal hepatic failure [101]. Studies in non-human primates and piglets demonstrated that intravenous injection of AAV vectors expressing human *SMN* at doses of 2 × 10^14^ GC/kg or above resulted in severe hepatotoxicity and dorsal root ganglion (DRG) degeneration, regardless of capsid serotype or transgene identity, suggesting these adverse events may represent a dose-dependent class effect intrinsic to high-dose AAV administration rather than to any specific vector design [102].

A single-patient DMD trial using intravenous rAAV9 at 1.0 × 10^14^ vg/kg resulted in fatal cardiopulmonary arrest eight days post-treatment, with the leading hypothesis implicating cytokine-mediated capillary leak syndrome [103]. High-dose intrathecal or intravenous AAV administration has been correlated with dorsal root ganglia (DRG) toxicity in both non-human primates and clinical subjects, presenting as axonal degeneration and neuronal injury, accompanied by lymphocytic infiltrates. Both direct transgene toxicity and capsid-specific T cell responses have been identified as contributing mechanisms [96,102].

### 7.4. Genotoxicity

Although rAAV genomes persist predominantly as episomal concatemers, integration into the host genome does occur and has been associated with hepatocellular carcinoma in multiple animal models [104,105]. Animal age and pre-existing liver disease appear to predispose toward tumour development in preclinical settings, though a direct causal relationship in human tumorigenesis has not yet been established [105,106]. When AAV is combined with CRISPR-based genome editing, the risk of vector integration is further elevated at sites of CRISPR-induced double-strand breaks, compounding the genotoxic liability of the combined approach [107,108].

### 7.5. Transgene Expression Durability and Promoter Silencing

Strong ubiquitous promoters such as CMV and CAG remain widely used, despite evidence that the CMV enhancer is susceptible to CpG dinucleotide methylation over time, which downregulates transgene expression both in vitro and in vivo [109,110]. Overexpression driven by strong synthetic promoters may also compete with endogenous gene expression, compromising cellular homeostasis and triggering transgene clearance [91,109]. Protective regulatory elements such as CBX3-UCOE (ubiquitous chromatin opening element) have been shown to prevent methylation-dependent silencing in iPSCs and their differentiated derivatives [88]. However, the practical utility of UCOE elements in AAV vectors is constrained by packaging capacity. The full-length UCOE element is approximately 1.5 kb, which, when combined with a promoter, coding sequence, and polyadenylation signal, substantially narrows the available cargo space within the 4.7 kb ssAAV limit. For transgenes already approaching this limit, incorporating a UCOE element may require truncation of either the UCOE itself or the therapeutic coding sequence, potentially compromising both silencing resistance and therapeutic efficacy. This packaging-silencing trade-off has not been systematically evaluated and represents a practical barrier to the implementation of UCOE-based stabilization strategies in AAV gene therapy. A distinct but related durability problem arises from passive episomal dilution in actively dividing cell populations. Episomal rAAV genomes are subject to passive dilution with each cell division [111,112]. In the liver, for example, hepatocytes undergo continuous turnover with an estimated individual cell lifespan of approximately three years [113]. Consequently, rAAV episomal genomes are progressively diluted during hepatocyte renewal, resulting in declining therapeutic transgene expression over time—a limitation observed in long-term clinical follow-up of haemophilia gene therapy trials [111,114].

### 7.6. Barriers to Re-Administration

A critical limitation of AAV-based gene therapy is that transgene expression has been observed to decline over multi-year follow-up in haemophilia and other trials, making re-administration clinically necessary in many therapeutic contexts [13,95]. However, a single AAV administration elicits durable neutralizing antibody (NAb) responses that persist long-term, effectively blocking repeat dosing with the same or a cross-reactive serotype in most systemic delivery contexts [94,96]. This creates a fundamental clinical paradox: the patients most likely to need a repeat dose are precisely those whose immune systems are most primed to neutralize it.

Three broad strategies are currently under investigation to overcome this barrier. The first aims to directly eliminate pre-existing or treatment-induced antibodies from circulation—either by physical removal through plasmapheresis, or by enzymatic degradation of IgG antibodies using IdeS/Imlifidase prior to vector administration [115,116]. The second approach focuses on shielding the vector from immune recognition, most notably by encapsulating AAV particles within extracellular vesicles, which physically conceal the capsid surface from circulating antibodies [35]. The third strategy involves engineering capsid variants that are sufficiently divergent from naturally occurring serotypes to evade recognition by pre-existing NAbs while retaining transduction efficiency [96]. The majority of these approaches remain in preclinical or early-phase clinical evaluation, and none has yet established a clear path to routine clinical re-dosing [35,115,116].

### 7.7. Manufacturing, Regulatory, and Clinical Translation Challenges

The clinical translation of AAV-based regenerative therapies introduces a set of challenges that are distinct from, but compounded by, the biological limitations discussed above. AAV manufacturing at clinical scale remains technically demanding: the ratio of genome-containing to empty capsids is a critical quality attribute that is difficult to control consistently across production batches, and empty capsids compete with genome-containing particles for cell-surface receptors, potentially reducing effective transduction at a given total dose [9,10]. Recent advances in closed bioreactor platforms have begun to address the scalability problem: a semi-automated hollow-fiber bioreactor system produced AAV2 at yields of 4.92 × 10^14^ viral particles from 1.2 L of cell lysate while reducing open manufacturing steps by more than 40-fold and production time by up to 7.5-fold compared to standard flask-based approaches, representing a significant step toward cost-effective, consistent clinical-grade production [117]. Residual manufacturing impurities, including host-cell proteins, residual baculovirus components in insect cell-derived preparations, and free DNA, represent additional variables that affect immunogenicity and are subject to evolving regulatory scrutiny.

Dose selection for AAV gene therapies remains empirical and is complicated by the absence of standardized potency assays that translate reliably across species. The doses used in clinical trials have been extrapolated from preclinical data in mice and non-human primates, but as the toxicity events in the ASPIRO and DMD trials demonstrate, the immunological and physiological response to high-dose AAV in humans is not fully predicted by preclinical models [101,102]. Regulatory agencies now require long-term monitoring of AAV-treated patients for integration events, immune-mediated transgene loss, and delayed genotoxicity, creating a post-approval surveillance burden that has not been uniformly implemented across approved products [9]. Patient selection criteria, including pre-existing NAb titres, immune status, and age, are increasingly recognized as determinants of both efficacy and safety, but standardised screening protocols have not yet been adopted industry-wide. These manufacturing and regulatory dimensions are central to whether AAV-based regenerative therapies can be scaled beyond individual academic trials to accessible, consistent clinical products.

## 8. Future Directions

Three priorities follow directly from the limitations documented in this review and should define the next phase of AAV research in regenerative and reprogramming contexts.

First, integration site analysis should be adopted as a mandatory safety endpoint for any iPSC-derived cell therapy developed using AAV vectors. The current practice of describing AAV as integration-free without empirical verification is inconsistent with the evidence from Senís et al. (2018) and Weltner et al. (2012) [51,52], both of which detected integration in every examined iPSC clone generated under reprogramming conditions. Routine integration profiling, analogous to the insertion site analysis already required for lentiviral gene therapies, should replace the assumption of non-integration as a default characterization of AAV in proliferating cell contexts.

Second, Cre-lox lineage tracing or an equivalent orthogonal validation strategy should be treated as a minimum experimental standard for any in vivo reprogramming claim made using AAV delivery. The scale of false-positive reprogramming signals documented by Puglisi et al. (2024) and Xie et al. (2023) [58,59], where promoter leakage into pre-existing neurons generated apparent conversion efficiencies of up to 80.2%, demonstrates that reporter expression alone is insufficient evidence of genuine cell fate change. Until lineage tracing becomes the norm rather than the exception, the published efficiency figures for in vivo glia-to-neuron conversion and similar reprogramming paradigms cannot be taken at face value.

Third, claims of cell-type-specific AAV delivery should require direct empirical demonstration in the target tissue rather than inference from capsid tropism data or promoter precedent. The consistent failure of both engineered capsids and cell-type-restricted promoters to achieve true cellular exclusivity, documented across CNS, cardiac, retinal, and pulmonary contexts in this review, means that specificity must be validated independently for each new vector–promoter–transgene combination. No precedent established with one transgene under a given promoter can be assumed to hold for a different transgene under the same promoter. Taken together, these three standards, integration profiling, lineage tracing, and empirical specificity validation, represent a minimum framework for rigorous AAV-based reprogramming research and should be considered prerequisites for clinical translation of any AAV-mediated cell therapy.

## 9. Conclusions

AAV has achieved genuine clinical translation, with approved therapies for SMA, hemophilia B, and retinal dystrophy demonstrating that sustained, therapeutically meaningful transgene expression is achievable in humans. The capsid engineering advances reviewed here, directed evolution, serotype screening, synthetic ITR variants, represent real progress toward overcoming the platform’s intrinsic constraints of packaging capacity, immunogenicity, and off-target biodistribution. Yet the same advances consistently unmask new trade-offs: improved tropism demands higher doses, dose escalation amplifies toxicity, and specificity gains in one tissue increase off-target exposure in another.

No current capsid or promoter combination achieves cell-type exclusivity within any target organ, a limitation with direct consequences for reprogramming applications, where the systematic failure of *GFAP*-driven promoters with neurogenic payloads has generated false-positive conversion signals in a substantial proportion of published in vivo studies [58,59].

Ultimately, the future of AAV-mediated regenerative medicine will depend not only on improving vector performance, but also on improving the precision with which vector-derived biological effects are interpreted. The next generation of AAV technologies should therefore be designed not merely to maximize transduction efficiency, but to achieve predictable, cell type-specific, and experimentally verifiable gene delivery. Only by combining advances in vector engineering with rigorous experimental validation can AAV fulfill its full potential as a platform for both regenerative biology and clinical gene therapy.

## Figures and Tables

**Table 2:** Performance comparison of engineered AAV capsid variants across experimental species and tissue contexts.

Variant	Base Serotype	Engineering Strategy	Primary Target Tissue	Species Tested	Key Advantage	Key Limitation	Reference
PHP.eB	AAV9	Directed evolution (CREATE)	CNS (broad, systemic)	Mouse; dramatically reduced in NHP	>90% CNS neuron transduction after IV in mice; non-invasive	Species-specific receptor dependence; poor NHP/primate efficacy limits clinical translation	Chan et al., 2017 [27]; Chatterjee et al., 2022 [31]
MyoAAV	AAV9	Directed evolution	Skeletal muscle	Mouse (primate data limited)	Reduced liver sequestration vs. AAV9; increased muscle transduction	6.3-fold elevated cardiac transduction vs. AAV9; age-, dose-, and background-dependent specificity	Tabebordbar et al., 2021 [28]; Ji et al., 2024 [32]
AAV2-7m8	AAV2	Rational capsid modification	Retina (photoreceptors, RPE)	Mouse; iPSC-derived organoids; NHP retina	64% photoreceptor transduction in organoids vs. 2.5% for AAV9	Receptor-dependent; *FGF2* in culture medium inhibits transduction via *FGFR1*	Garita-Hernandez et al., 2020 [29]
rAAV.KK04	AAV6	Directed evolution in hiPSC-CMs	Cardiomyocytes	Human (iPSC-derived; cardiac slices)	Outperforms AAV6 in 2D cultures, cardiac organoids, and human cardiac slices	High MOI required; potentiates DDR/p53/STING axis; not yet tested in vivo	Kok et al., 2023 [30]
AAVv66	NHP isolate	Naturally occurring variant	CNS (broad distribution)	Mouse; NHP	Improved manufacturing yield; broader CNS biodistribution; partial NAb evasion	Limited clinical data; not yet in human trials	Hsu et al., 2020 [20]
AAV-DJ	Chimeric (AAV2/8/9)	Capsid shuffling	Multiple (primarily in vitro)	Human primary cells; iPSCs	High in vitro efficiency; heparin-binding domain from AAV2	Broad tropism; limited cell-type specificity; primarily used in research settings	Drouin & Agbandje-McKenna, 2014 [14]

NHP, non-human primate; IV, intravenous; hiPSC-CM, human iPSC-derived cardiomyocyte; NAb, neutralising antibody; DDR, DNA damage response; MOI, multiplicity of infection; RPE, retinal pigment epithelium; CREATE, *Cre* recombination-based AAV targeted evolution.

**Table 3:** Major Limitations of rAAV Vectors: Mitigation Strategies, Remaining Barriers, and Translational Status.

Category/Limitation	Consequence	Current Mitigation Strategy	Remaining Barrier	Translational Status
Packaging capacity (~4.7 kb ssAAV; ~2.5 kb scAAV)	Excludes full-length *dystrophin* (~11.4 kb) and *CEP290* (~7.5 kb); scAAV efficiency advantage restricted to small-cargo applications	Transgene truncation (microdystrophin); dual-vector reconstitution at DNA/RNA/protein level	Truncated proteins of uncertain functional equivalence; dual-vector co-delivery efficiency variable	Microdystrophin in Phase III (*DMD*); no full-length solution for *CEP290*
Episomal dilution in dividing cells	Non-replicating episomes passively diluted with each cell division; fundamental incompatibility with iPSC proliferation	Serial re-transduction; scAAV for faster expression onset	Dilution intrinsic to non-replicating episomes; no solution without integration	Unresolved; not yet addressed clinically
Promoter silencing (CpG methylation)	CMV, EF1α, CAG, and synthetic promoters susceptible to methylation over time; largely irreversible in iPSCs	UCOE elements; synthetic promoters; tissue-specific promoters	UCOE adds ~1.5 kb cargo burden; silencing irreversible once established	Preclinical; UCOE not yet in clinical AAV products
Pre-existing neutralising antibodies (NAbs)	Seroprevalence up to 90% for some serotypes; even low titres abolish transduction	Serotype switching; plasmapheresis; IdeS/Imlifidase; EV-AAV shielding	Cross-reactivity limits switching; enzymatic strategies add complexity	IdeS in Phase I/II; plasmapheresis clinical use
Capsid-specific CD8^+^ T cell responses	Dose-dependent; causes transaminitis and transgene loss in clinical trials	Immunosuppression (corticosteroids); empty capsid decoys; capsid engineering	T cell responses dose-dependent; immunosuppression increases clinical risk	Standard of care in high-dose trials; no preventive solution
Barriers to re-administration	Durable NAbs after single dose block repeat dosing with same or cross-reactive serotype	EV-AAV; alternative serotypes; immune tolerance induction	No established clinical re-dosing protocol; NAb responses durable for years	Preclinical/early phase
High-dose hepatotoxicity and DRG degeneration	Fatal events in ASPIRO and DMD trials; class effect at ≥2 × 10^14^ GC/kg	Lower dose + efficient capsids; regional delivery; dose fractionation	Therapeutic and toxic doses may overlap for some indications	Active regulatory review; ASPIRO high-dose cohort halted
Genotoxicity (integration; HCC risk)	Rare integration events linked to HCC in animal models; CRISPR co-delivery elevates risk	Integration site analysis; avoidance of strong enhancers near proto-oncogenes	No clinical HCC causally confirmed; CRISPR compounding risk not yet quantified	Mandatory long-term follow-up in approved products
iPSC cytotoxicity (ITR G-quadruplex; p53 apoptosis)	AAV genome triggers p53-dependent apoptosis selectively in pluripotent state; empty capsids non-toxic	Synthetic ITR variants; *p53*/*STING* small-molecule inhibitors	Safety-efficacy trade-off with modified ITRs; inhibitors not clinically validated	Preclinical
Liver sequestration (up to 90%) of systemic dose	Payload diverted to liver regardless of intended target; requires 100–250× higher doses for muscle vs. engineered variants	Myotropic/neurotropic engineered capsids; local/regional delivery	Organ-level detargeting does not resolve cell-type specificity within organ	MyoAAV preclinical; no approved detargeted capsid
Lack of cell-type specificity within organs	All tested serotypes transduce multiple cell types in CNS, heart, retina, and liver; no capsid achieves cellular exclusivity	Cell-type-specific promoters; synthetic enhancers; combinatorial approaches	No serotype achieves exclusive single-cell-type transduction in any organ	Unresolved; empirical validation required per construct
Promoter infidelity with neurogenic payloads	*GFAP* promoter faithfully restricts mCherry to glia but drives neurogenic factors (*NeuroD1*, *Ascl1*, *Math5*) predominantly in neurons—insert-dependent failure generating false-positive reprogramming signals in up to 80.2% of HuC/D+ cells [59]	Cre-lox lineage tracing; miR-124 target sequences in 3′ UTR	Insert-dependent failure unpredictable a priori; miR approach reduces expression level	Standard not yet adopted in field

HCC, hepatocellular carcinoma; DRG, dorsal root ganglion; NAb, neutralising antibody; UCOE, ubiquitous chromatin opening element; EV-AAV, extracellular vesicle-encapsulated AAV; iPSC, induced pluripotent stem cell; scAAV, self-complementary AAV; ssAAV, single-stranded AAV; CRISPR, clustered regularly interspaced short palindromic repeats; GC/kg, genome copies per kilogram.

## Data Availability

No new data were created or analyzed in this study. Data sharing is not applicable to this article.
